# Ongoing mpox outbreak in Kamituga, South Kivu province, associated with monkeypox virus of a novel Clade I sub-lineage, Democratic Republic of the Congo, 2024

**DOI:** 10.2807/1560-7917.ES.2024.29.11.2400106

**Published:** 2024-03-14

**Authors:** Leandre Murhula Masirika, Jean Claude Udahemuka, Leonard Schuele, Pacifique Ndishimye, Saria Otani, Justin Bengehya Mbiribindi, Jean M. Marekani, Léandre Mutimbwa Mambo, Nadine Malyamungu Bubala, Marjan Boter, David F. Nieuwenhuijse, Trudie Lang, Ernest Balyahamwabo Kalalizi, Jean Pierre Musabyimana, Frank M. Aarestrup, Marion Koopmans, Bas B. Oude Munnink, Freddy Belesi Siangoli

**Affiliations:** 1 Centre de Recherche en Sciences Naturelles de Lwiro, DS Bukavu, South Kivu, Bukavu, Democratic Republic of the Congo; 2 SaBio Instituto de Investigación en Recursos Cinegéticos IREC (Universidad de Castilla-La Mancha & CSIC), Ciudad Real, Spain; 3 Department of Veterinary Medicine, University of Rwanda, Nyagatare, Rwanda; 4 Stansile Research Organization, Kigali, Rwanda; 5 Department of Viroscience, Erasmus University Medical Center, Rotterdam, The Netherlands; 6 Department of Microbiology and Immunology, Dalhousie University, Halifax, Nova Scotia, Canada; 7 Research and Innovation Centre, African Institute for Mathematical Sciences, Kigali, Rwanda; 8 Research Group for Genomic Epidemiology, National Food Institute, Technical University of Denmark, Kongens Lyngby, Denmark; 9 Hôpital Général de Référence de Kamituga, South Kivu, Bukavu, Democratic Republic of the Congo; 10 Unit of Animal Production and Health, Nature Conservation and Development, Department of Biology, Faculty of Science, University of Kinshasa, Kinshasa, Democratic Republic of the Congo; 11 Zone de Santé de Kamituga, Kamituga, South Kivu, Bukavu, Democratic Republic of the Congo; 12 The Global Health Network, Oxford University, Oxford, United Kingdom; 13 Division Provinciale de la Santé, South Kivu, Bukavu, Democratic Republic of the Congo; 14 Research, innovation and data science division, Rwanda Biomedical Center, Kigali, Rwanda; * These authors contributed equally

**Keywords:** monkeypox virus, mpox, genomics, surveillance, Democratic Republic of the Congo

## Abstract

Since the beginning of 2023, the number of people with suspected monkeypox virus (MPXV) infection have sharply increased in the Democratic Republic of the Congo (DRC). We report near-to-complete MPXV genome sequences derived from six cases from the South Kivu province. Phylogenetic analyses reveal that the MPXV affecting the cases belongs to a novel Clade I sub-lineage. The outbreak strain genome lacks the target sequence of the probe and primers of a commonly used Clade I-specific real-time PCR.

In the Democratic Republic of the Congo (DRC), the numbers of people with suspected infection with monkeypox virus (MPXV), the virus that causes mpox, have increased since the start of 2023. A total of 12,569 suspected mpox cases have been reported up to 12 November, the highest number of annual cases ever recorded [1]. The case fatality rate has been estimated at 4.6% [1]; moreover, new cases have occurred in geographical areas of the country where the disease was previously not observed, such as Kinshasa and South Kivu province [1,2]. Despite this concerning situation, there is only limited genomic information available on the circulating viruses, which suggests that they belong to Clade I [3]. To gain more insight into the characteristics of the strains causing the epidemic, as well as assurance that current and commonly used molecular assays to diagnose MPXV infections can detect these strains, we sequenced monkeypox viral genomes from recently diagnosed cases in South Kivu, DRC.

## Case definitions and patient characteristics

A case was listed as ‘suspect’ if presenting with an acute illness with fever, intense headache, myalgia, and back pain, followed by 1 to 3 days of a progressively developing rash often starting on the face and spreading on the body. A confirmed mpox case had a monkeypox virus infection which was laboratory-confirmed by PCR. A case was listed as ‘probable’ when satisfying the clinical definition of a suspected case and having an epidemiological link to a confirmed or probable case; a probable case was not laboratory-confirmed [4].

The study involved patients from South Kivu province in the territory of Mwenga, who were hospitalised in the Kamituga hospital, which is in the Kamituga health zone. The first mpox cases in this area were reported from September 2023 onwards.

A total of 10 patients were included in the study. All were young adults in their late teenage up to the age of mid-20 years and five were male and five females. Regarding professions comprised, the majority of the concerned individuals were sex workers. For these patients, admission to the Kamituga Hospital had been based on clinical diagnosis of mpox by hospital staff. According to routines, skin lesion and oropharyngeal swabs collected from the patients had been sent to the national medical research institute of the DRC (Institut National de la Recherche Biomédicale; INRB) in Goma for MPXV infection confirmation by PCR, and all patients had tested positive for the virus.

## DNA library preparation, sequencing and phylogenetic analysis

The samples from the 10 patients had been collected throughout the month of January 2024, stored in virus transport medium and frozen at −20 °C. For sequencing, DNA was extracted with the Blood and Tissue Kit (Qiagen) according to the manufacturer’s recommendations. Viral transport medium was used as a negative control. MPXV DNA was amplified using a mpox amplification scheme specifically designed for Clade IIb MPXV to generate 2,500 bp amplicons and quantified using the Qubit dsHS DNA assay (ThermoFisher) [5]. Sequencing libraries were prepared using the Native Barcoding Kit 24 V14 (SQK-NBD114.24, Oxford Nanopore Technologies; ONT) using a R10 flow cell.

Sequencing reads were basecalled using dorado v.7.2.13 with the super-accurate base calling model (ONT). Primer sequences were removed using cutadapt v4.6 [6] and aligned against a MXPV reference (GenBank accession: OQ729808) in MinKNOW v23.11.5 (ONT). The consensus was created from the resulting bam file using samtools v1.19.2 as described [7,8]. NextClade v3.1.0 was used for clade assignment and quality checks [9].

Sequences were aligned using MAFFT v7.520 [10], and the alignment manually curated. Phylogenetic analysis was performed using IQ-TREE v2.2.6 [11], visualised with FigTree v1.4.4 [12], and for additional confirmation of the results, phylogenetic analysis was also performed using NextStrain CLI v8.2.0 and visualised in auspice.

## Identification of monkeypox viruses belonging to novel Clade I sub-lineage

At the time of writing, no near-complete sequences of MPXV circulating in DRC from the 2023 outbreak were available on (public) online databases. The targeted amplicon sequencing conducted to characterise MPXV sequences in the 10 hospitalised study patients allowed to generate near-complete MPXV sequences for six of them; all were classified as Clade I. The genome coverage ranged between 93.5%–100% (average of 95.2%) as described in **Supplementary Tables 1** and **2** .

**Figure 1:**
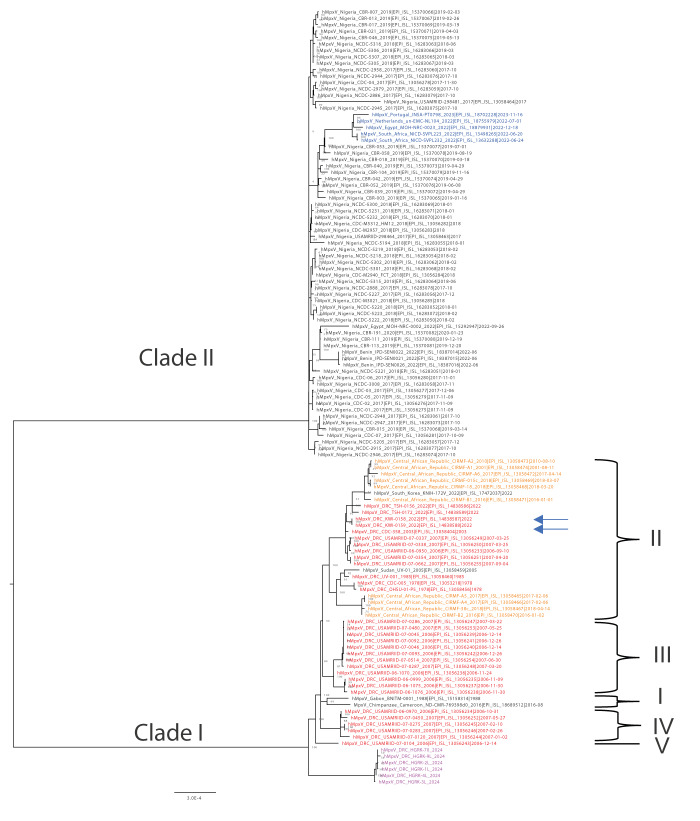
Phylogenetic analysis of all near-to-complete genome mpox sequences from Africa available on GISAID and the NCBI including the 2024 sequences from Kamituga, Democratic Republic of the Congo (n = 121 sequences)

DRC: Democratic Republic of the Congo; NCBI: National Center for Biotechnology Information.

Phylogenetic analysis was done including 113 near-to-complete genome reference mpox sequences from Africa available from GISAID [13], which include all sequences available on the National Center for Biotechnological Information (NCBI) on 8 February 2024 and two European sequences from the recent 2022 global mpox outbreak [14]. The six sequences from the current study grouped with published Clade I sequences, but in a distinct sub-lineage from all other Clade I sequences, suggesting that the ongoing outbreak in South Kivu results from a separate introduction (**Figure 1** and **Supplementary Figure 1** and **2** ). The six sequences have several single nucleotide polymorphism (SNP) differences between them, which suggests ongoing circulation of this outbreak strain for some time already.

The scale under the tree represents the number of substitutions per site. Sequences from Kamituga are presented in magenta, previous sequences from DRC in red and sequences from the 2022 outbreak in blue [14]. Arrows indicate sequences which have been shown in a prior publication to cluster with the previous sequences from a 2023 MPXV outbreak from Kwilu Province near Kinshasa [3]. Lineages are indicated as proposed by Nakazawa et al. [19] and Berthet et al. [20].

We gratefully acknowledge all data contributors, i.e., the authors and their originating laboratories responsible for obtaining the specimens, and their submitting laboratories for generating the genetic sequence and metadata and sharing via the GISAID Initiative, on which this research is based.

## The monkeypox virus outbreak strain in South Kivu lacks the target sequence used for identifying Clade I viruses

**Figure 2:**
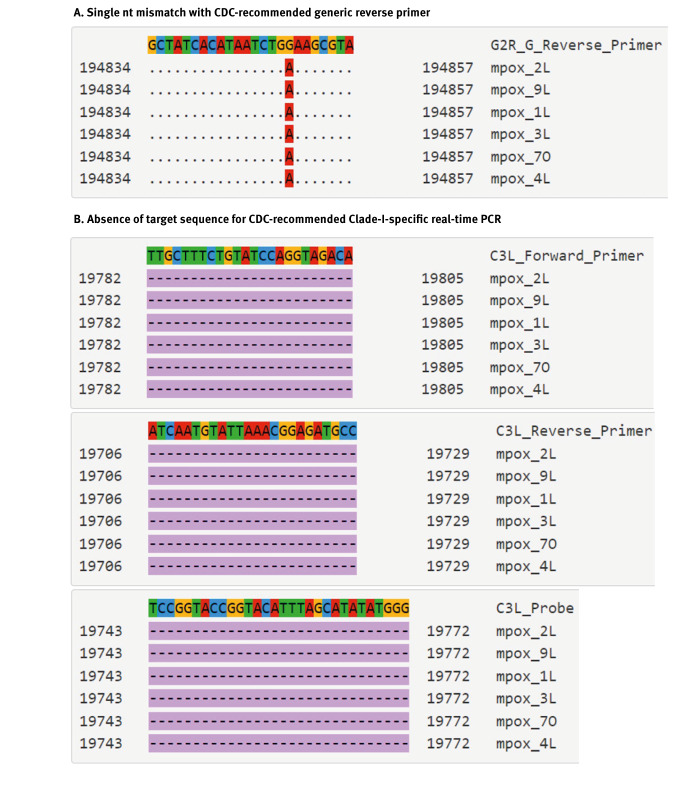
Sequence alignments highlighting genetic sequence features of the MPXV detected in Kamituga resulting in (A) a single nt mismatch with US CDC recommended generic reverse primer (G2R_G reverse primer) and (B) absence of the US CDC recommended clade I specific forward, reverse, and probe target location (C3L), Democratic Republic of the Congo, 2024

To check if the strains obtained in the current study could be detected by commonly used molecular assays to diagnose MPXV infections, their sequences were aligned to the closely related Clade I sequence EPI_ISL_13056243. This sequence matches primer and probe sequences recommended by the United States (US) Centers for Disease Control and Prevention (CDC) to diagnose MPXV [15]. The alignment was assessed using an in-house Primer Check Tool (https://viroscience-emc.shinyapps.io/primer-check/ ). While the generic primers and probe still seem to be functional with only one mutation in the reverse primer, the specific Clade I virus real-time PCR target, recommended by the US CDC, is absent in the genomes of the novel MPXV strains (**Figure 2** and Supplementary Figure 3). The observed deletion is 1,114 nt in size and results in the complete deletion of the OPG032 gene. The coverage of this region ranged between 76× and 941× sequence reads depending on the sample. Due to the deletion, the rapid US CDC method to identify Clade I in newly diagnosed mpox cases is most likely not reliable for detection of the novel sub-lineage identified in the current study.

US CDC: United States Centers for Disease Control and Prevention; MPXV: monkeypox virus.

The sequence matching the US CDC primers or probe annealing sites is on the top line, where each nt is highlighted in a different colour. The lines underneath the top line each represent sequences of the MPXV identified in the current study, which are aligned to the sequence on the top line. At each position of a study sequence, identity to the top sequence is represented by a dot. When a difference is a substitution, the nt present in each study sequences is indicated, when a difference is a deletion, this is represented by a dash highlighted in purple.

Visualised by primer-check tool: https://viroscience-emc.shinyapps.io/primer-check/.

## Discussion

MPXV is an emerging zoonotic virus belonging to the *Poxviridae* family and the genus *Orthopoxvirus*. In the past, MPXV has been primarily detected in West and Central Africa, however, in 2022 the World Health Organization (WHO) declared a multi-country global outbreak of MPXV of Clade IIb [14,16]. This outbreak was predominantly described as affecting communities of men having sex with men (MSM), although infections were also reported in a limited number of people not engaging in sexual contact with infected individuals.

In DRC, where human infection with MPXV was first ever recognised in 1970, sporadic mpox outbreaks have been reported since, with increasing frequency over time [17]. The particular rise in the number of suspected MPXV cases recorded in the country in 2023, and their occurrence in unusual geographic areas [2] suggests a possible change in the characteristics of the virus and the epidemiology of the disease. Therefore, obtaining sequence information on the circulating strain(s) is considered crucial and MPXV whole genome Nanopore sequencing training has been provided recently to local scientists from Rwanda and DRC to perform both wet-laboratory sequencing and dry-laboratory sequence analysis.

From the mpox outbreak in Kamituga, South Kivu, six near-to-complete MPXV sequences derived from local patients hospitalised with mpox were obtained. Phylogenetic analyses of these sequences together with those available for other Clade I and II viruses, placed them in a new sub-lineage near the root of Clade I, which suggests that the outbreak in this region results from a new introduction, most likely from a zoonotic reservoir. Although sequences from a small 2023 Kinshasa outbreak are not publicly available, the placement of those sequences in a published phylogenetic tree [3] suggests that the Kamituga outbreak is not related to the outbreak in Kinshasa. Our findings therefore suggest that there are at least two independent outbreaks ongoing in DRC.

Remarkably, a large stretch of sequence in the genomes belonging to the novel MPXV sub-lineage was absent compared to other Clade I genomes, which would lead to failure of the Clade I-specific real-time PCR recommended by the US CDC [15]. A deletion in the same region is also observed in Clade II MPXV, and this was the basis for clade assignment using the CDC PCR. Therefore, if the viruses from the new lineage were to spread internationally, this molecular surveillance tool can no longer be used to rapidly identify these Clade I virus infections while the global Clade IIb outbreak is ongoing.

Multiple amplicon-based assays targeting Clade IIb MPXV have been developed and applied since the global mpox outbreak in 2022 [5,18]. In the current work, the application of a large amplicon assay which was originally designed to target Clade IIb resulted in an average genome coverage of 95.2% in the new Clade I genome, showing that this method can also be used to sequence Clade I viruses, although minor adjustments of primer pairs might result in more complete consensus sequences. Also, the consensus was generated using a reference-based alignment using Nanopore data. Metagenomic Nanopore or Illumina sequencing might be needed in combination with de novo assembly approaches for refined consensus calling, however, this was not possible here, making this a limitation of the current work. Further studies are needed/ongoing to assess transmissibility and clinical severity associated with the new lineage.

### Conclusion

Altogether, the findings of this study strongly suggest that whole genome sequencing of a larger subset of MPXV currently causing mpox cases in DRC, as well as public data sharing, are essential to understand the ongoing epidemic. Further studies, sequencing and analyses are ongoing, but in accordance with the above statement we believe that rapid public sharing of all available information is essential to help to better understand and contain the current mpox emergence.

## Supplementary Data

1141000 Supplementary Material
