# Authors’ correction for Euro Surveill. 2024;29(11)

**DOI:** 10.2807/1560-7917.ES.2024.29.12.240321c

**Published:** 2024-03-21

**Authors:** Eurosurveillance editorial team

**Affiliations:** 1 European Centre for Disease Prevention and Control (ECDC), Stockholm, Sweden

In the article entitled ‘Ongoing mpox outbreak in Kamituga, South Kivu province, associated with monkeypox virus of a novel Clade I sub-lineage, Democratic Republic of the Congo, 2024’ by Masirika et al., published on 14 March 2024, some affiliation numbers in the authors’ list were incorrect for the following authors: Justin Bengehya Mbiribindi, Jean Pierre Musabyimana and Freddy Belesi Siangoli. Affiliation numbers were corrected on 18 March 2024.

