# Supplementary material for: Ongoing mpox outbreak in Kamituga, South Kivu province, associated with monkeypox virus of a novel Clade I sub-lineage, Democratic Republic of the Congo, 2024
Source: Euro Surveill. 2024 Mar 14;29(11):2400106. doi: 10.2807/1560-7917.ES.2024.29.11.2400106 (PMC10941309; doi:10.2807/1560-7917.ES.2024.29.11.2400106)
Supplement: Supplementary Material [file 2400106_SupplementaryMaterial.pdf]

This supplementary material is hosted by *Eurosurveillance* as supporting information alongside the article [**Ongoing mpox outbreak in Kamituga, South Kivu province, associated with monkeypox virus of a novel Clade I sub-lineage, Democratic Republic of the Congo, 2024**], on behalf of the authors, who remain responsible for the accuracy and appropriateness of the content. The same standards for ethics, copyright, attributions and permissions as for the article apply. Supplements are not edited by *Eurosurveillance* and the journal is not responsible for the maintenance of any links or email addresses provided therein."

Supplementary Table 1: Clade assignment and quality metrics of mpox consensus sequences.

Sample ID	Clade[a]	Genome coverage	Coverage depth
1 L	I	100%	6477.1x
2 L	I	93.5%	959.2x
3 L	I	94.3%	2420.6x
4 L	I	94%	2950.0x
7 O	I	93.8%	3186.3x
9 L	I	95.3%	1709.9x

[a] Clade assignment as defined by Nextclade v3.1.0. **Abbreviations:** L, Lesion (skin); O, oropharyngeal swab.

Supplementary Table 2: Clade assignment and sequence quality of 10 sampled patients with suspected mpox infection.

Patient ID	Clade[a]	Skin lesion swab		Oropharyngeal swab	
		Genome coverage	Coverage depth	Genome coverage	Coverage depth
1	I	100%	6477.1x	94.2%	2357.8x
2	I	93.5%	959.2x		
3	I	94.3%	2420.6x		
4	I	94%	2950x	86.2%	27.9x
5	I	90.9%	79x		

6	I				
7	I	93.5%	1232.3x	93.8%	3186.3x
8	I	94.2%	3214.5x		
9	I	95.3%	1709.9x		
10	I	91.4%	2866.2x		

[a] Clade assignment as defined by Nextclade v3.1.0; Empty fields indicate no mpox amplicon product.

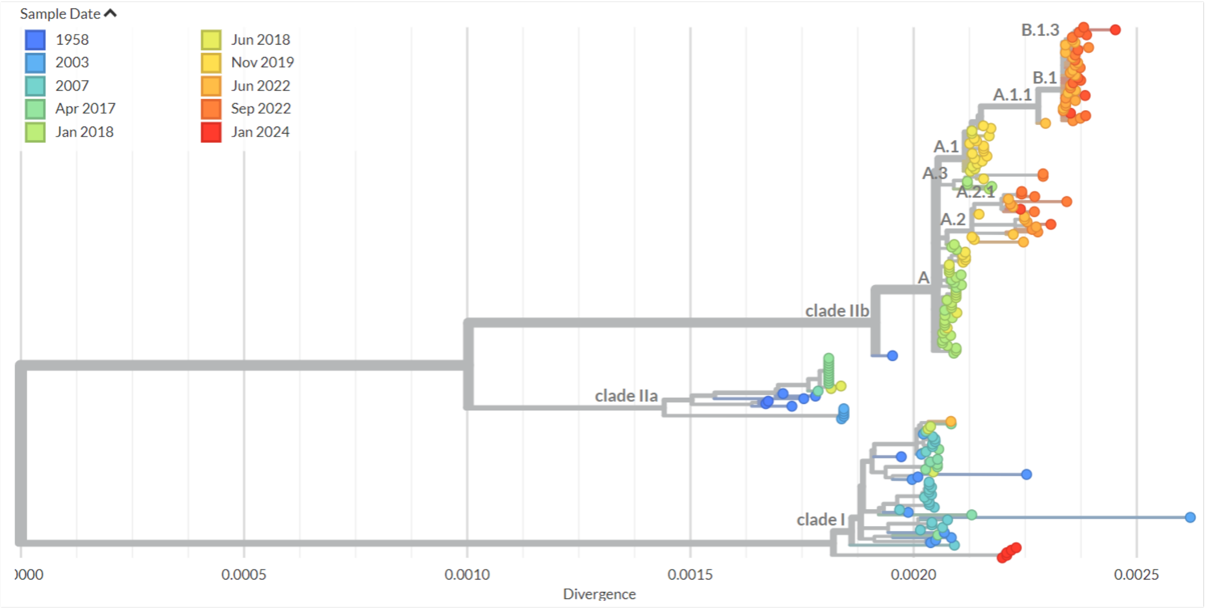

Supplementary Figure 1: Phylogenetic tree showing 211 mpox genomes including the six new sequences from Kamituga in dark red.

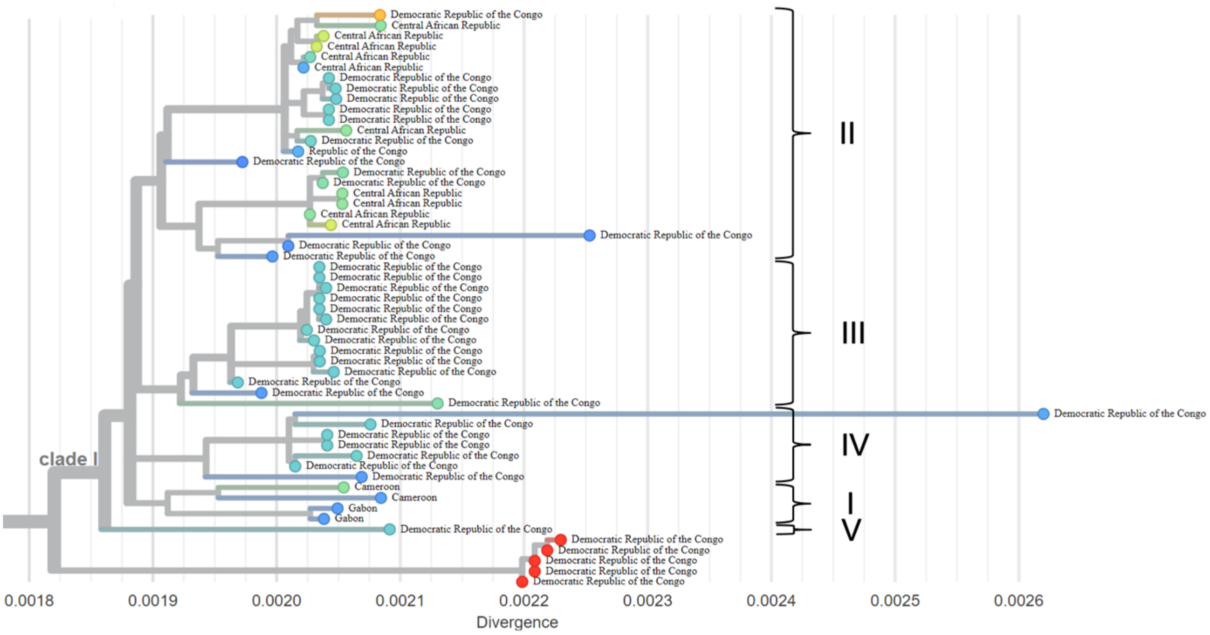

Supplementary Figure 2: Zoom of the phylogenetic tree focussing on the Clade I viruses. Sequences from this study are shown by enlarged red circles.

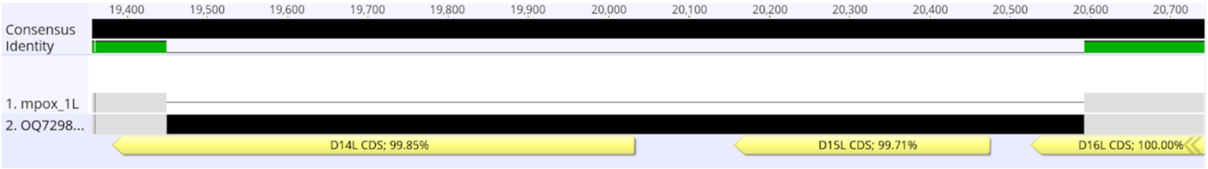

Supplementary Figure 3: Alignment of exemplary sequenced mpox sample and reference highlighting the deletion downstream of the 3' ITR (position 19,451-20,593).
